# Assessment of Jordanian health care professionals’ perception towards new COVID-19 variants of concern

**DOI:** 10.1371/journal.pone.0265797

**Published:** 2022-11-18

**Authors:** Hana M. Sawan, Shatha M. Al Omari, F. Al Bahar, Reema Karasneh

**Affiliations:** 1 Faculty of Pharmacy, Pharmaceutical Sciences Department, Zarqa University, Zarqa, Jordan; 2 Faculty of Pharmacy, Department of Clinical Pharmacy, Zarqa University, Zarqa, Jordan; 3 Faculty of Medicine, Department of Basic Medical Sciences, Yarmouk University, Irbid, Jordan; National Institute of High Security Animal Diseases, INDIA

## Abstract

**Background:**

Healthcare professionals working at the frontline, dealing with COVID-19 patients or their samples, should know about variants of concern (VOCs) and their transmissibility, disease severity, and vaccine efficacy. Healthcare professionals’ (HCPs) perceptions towards new VOCs affect their practice and attitudes towards their patients. Moreover, these perceptions might significantly impact their patients’ perceptions of new COVID-19 variants and public vaccine acceptability.

**Methods:**

Online and paper-based questionnaires were distributed among Healthcare professionals in Jordan between August 2021 and October 2021.

**Results:**

Among 423 HCPs who participated in this study, a majority believe that when viruses mutate, they become more transmissible (77.8%), more deadly (61.7%), and pathogenic (64.8%). In addition, half of the respondents, perceived current treatments as partially effective against VOCs and current diagnostics to be efficient. However, all VOCs were perceived as more transmissible, more virulent, and related to higher mortality rates when compared to the original strain. Regarding immunity against VOCs, (57.4%) of respondents believe in partial immunity against re-infection, and most respondents were either unsure about the current vaccines’ efficacy or agreed that available vaccines would be ineffective. However, respondents (44.4%) still believe that people previously infected should get vaccinated. Respondents referred to the Ministry of Health as the most reliable source of information (45.6%) and the party responsible for educating the public about COVID-19 VOCs (57.9%). Travel was not a source of worry among respondents. However, they were worried about their families getting the new COVID-19 VOCs from their work. Similar proportions agreed/disagreed on the efficacy of the precautions and infection control measures currently applied by the government for preventing the spread of the new COVID-19 VOCs.

**Conclusion:**

Campaigns, workshops, and webinars targeting vaccines are highly recommended among HCPs to increase public acceptance of the vaccine and further booster shots.

## Introduction

As of December 2020, new variants of COVID-19 were spotted in the UK and South Africa and resulted in a horrifying increase in the number of cases reported in these countries and sooner spread to other countries [1,2]. Besides concerns regarding the overburdened healthcare systems, the emergence of new variants has cast a shadow over socioeconomic aspects of life (e.g., international travel, fears of failure of reopening and going back to lockdown) and being responsible for multiple waves of COVID-19. Viruses evolve through two main mechanisms; antigenic shift and antigenic drift. COVID-19 emergence has been attributed to antigenic shift, while new variants have evolved through antigenic drift [3].

Fortunately, not all viral mutations are clinically significant. Those mutations involving spike protein genes can mainly result in the emergence of clinically distinct variants [4]. According to the Centers for Disease Control and Prevention (CDC), a variant of concern (VOC) is defined as “a variant for which there is evidence of an increase in transmissibility, more severe disease (e.g., increased hospitalizations or deaths), a significant reduction in neutralization by antibodies generated during previous infection or vaccination, reduced effectiveness of treatments or vaccines, or diagnostic detection failures” [5]. As of August 2022, one COVID-19 variant is regarded as a VOC by the WHO and the CDC [6]; i.e. Omicron (B.1.1.529). Alpha (B.1.1.7, UK, Kent), Beta (B.1.351, South Africa), Gamma (P1, Brazil), Delta (B.1. 617.2, India) variants were classified as VOCs at the beginning of this study and then were de-escalated.

Many studies have reported a higher transmission rate for VOCs, e.g., 30%-45% higher transmission in B.1.1.7 variant, especially in younger age groups [7,8], and 2.5 fold increase in transmissibility for the P1 variant [9]. This effect is attributed to D614G mutation in the viral spike (S) protein (spike (S) protein is responsible for receptor binding and viral entry to host cells). D614G mutation (detected in all variants expressing higher transmission), was also found to be involved in enhancing viral affinity for olfactory nerve epithelium, and higher viral load in upper airways in animal models [10–13].

Higher false-negative results of RT-PCR for VOCs were also reported due to primer binding site mutations, e.g., decreased diagnostic sensitivity of Alpha variant was observed when using specific commercial kits targeting the spike protein gene [14]. In addition, serological testing might also be affected in case of mutations causing epitope loss [15]. Furthermore, the biggest concern jeopardizing healthcare efforts is the ability of VOCs to escape antibodies produced naturally during infection or after vaccination. VOCs’ immune evasion was mediated through reduced cross-reactivity between VOCs and neutralising antibodies [16]. Moreover, several studies have demonstrated diminished vaccines efficacy against some VOCs due to RBD mutations [17], and it seems that the Beta variant is the most resistant VOC against different types of vaccines [18].

Therapeutics used for wild variants were also reported to be compromised by the emergence of VOCs, e.g., using a mouse model, convalescent plasma collected from a patient infected in early 2020 was found ineffective against Alpha and Beta variants [19]. In addition, some mutations have been reported to cause treatment failure of monoclonal antibodies, e.g., E484K mutation compromising bamlanivimab monotherapy and Q493R mutation compromising bamlanivimab/etesevimab combination therapy [20]. Finally, regarding immunity after primary infection, although still not fully elucidated, at least one study has suggested a link between scant antibody titre and loss of immune protection [21], although the immune response to COVID-19 depends on complex interactions between antibody-producing B cells and T cells rather than B cells alone [22].

## Aims and objectives

This study aims to explore the perceptions and attitudes of Jordanian health care professionals towards new variants of the coronavirus. In addition, in this study, we will examine the awareness of Jordanian health care professionals towards the new COVID-19 VOCs.

## Methods

### Questionnaire development

A self-administered questionnaire was developed based on related literature and several international studies [23,24]. An online and paper version of the questionnaire consisting of 25 questions was used to collect data. The first section of the questionnaire collected basic demographic information (10 questions). The second section consisted of Likert-scale questions focusing on healthcare professionals’ knowledge of new variants of COVID-19. The last section of the questionnaire consisted of questions focussing on the perceptions and attitudes of health care professionals towards new variants of COVID-19. Questions (13–19) have Likert-scale statements with (agree, disagree, neutral) options. The questionnaire tool was adapted using previously available questionnaires on the same subject with slight modifications related to the new variants of COVID-19. The tool was developed in Arabic, then translated into English, and piloted among five academics experienced in infectious diseases (the questionnaire was administered in both Arabic and English languages, giving respondents the opportunity to choose their preferred language of choice).

Representatives of healthcare professionals have been involved in the development of the questionnaire. Comprehension of the questionnaire by members of the healthcare professionals, to improve the quality of the questions, was done by piloting the English version of the questionnaire on ten individuals and the Arabic version on seven individuals. This allowed for spotting any ambiguity in words or phrases, and the questionnaire was modified accordingly. Data obtained from the pilot were not included in the data analysis.

### Study design and setting

This cross-sectional study collected information from Jordanian healthcare professionals on the perceptions and attitudes towards new VOCs of COVID-19.

### Study sample

Our study sample consisted of health care professionals in Jordan, including both medical and non-medical professionals. Our sample consisted of physicians, pharmacists, nurses, lab technicians, and others working in public or private hospitals. As the percentage of the country’s HCPs who received the survey was not known, we could not calculate the response rate. The target sample size was determined by an online sample size calculator (i.e., Raosoft) [25] as 385, considering the number of licensed health care professionals (i.e., Pharmacists, Physicians, and Nurses) in Jordan, according to the latest version of the National Human Resources for Health Observatory Annual Report [26], assuming 50% response rate and 95% confidence level (i.e. 5% accepted margin of error).

### Data collection

Paper-based (n = 150) and online questionnaires (n = 273) collected data between August 2021 and October 2021. The survey link was shared through social media and was emailed to healthcare professionals. In addition, paper copies of the questionnaire were printed and filled face to face by other health professionals in different health care centres. All participants were required to fill in a consent form (written or electronic) before filling in the questionnaire. To maximise confidentiality, personal identifiers were not required.

### Ethical approval

The study was approved by the Zarqa University Ethics Committee (approval # 1/6/2021) (S1 Appendix).

### Outcome measures

The following outcomes were assessed: participants’ knowledge, perceptions, attitudes, and dangers of these variants.

### Data management and analysis

Descriptive analysis was applied and categorical variables were described with frequencies and percentages.

## Results

A total of 423 healthcare professionals completed the questionnaire. More females were included in the study sample (67.8%) than males, most within the age group of 25–49 (93.1%). Nurses/ midwives were the most frequent health care professionals in the study (35.9%), followed by pharmacists (30.0%). Half of the participants have work experience of fewer than five years (with 65% holding a bachelor’s degree and 20% holding graduate degrees), 66.4% have either managed, screened, or treated COVID patients, and 77.5% had COVID-19 infection themselves or had an infected family member. The vast majority of respondents (91%) received or registered to receive the COVID-19 vaccine [Table 1].

**Table 1:** HCPs’ sociodemographic and background characteristics (n = 423).

Variable	Frequency (Percentage %)
**AGE**	
25–49	394 (93.1)
> 50 years	29(6.9)
**Your health care profession**	
Physician	65 (15.4)
Pharmacist	127 (30.0)
Nurse/midwife	152 (35.9)
Others (lab technician, researchers, …, etc.)	79 (19.6)
**Facility sector**	
Public	200(47.3)
Private	223(52.7)
**Work experience (years):**	
< 5 years	211(49.9)
≥ 5 years	212(20.8)
**Gender**	
Male	136(32.2)
Female	287(67.8)
**Residency**	
North	80 (18.9)
Middle	325 (76.8)
South	18 (4.3)
**The highest degree or level of education you have completed:**	
Diploma degree	65(15.4)
Bachelor’s degree	275(65.0)
Graduate studies	83 (19.6)
**Have you managed, screened, or treated any known COVID patient?**	
Yes.	281(66.4)
No.	142(33.6)
**Have you or any of your family members been infected with COVID-19:**	
Yes.	328(77.5)
No.	95(22.5)
**Have you received or registered to receive the COVID-19 vaccine?**	
Yes.	385(91.0)
No.	38(9.0)

Participants have shown fair amount of knowledge pertaining viral mutation and COVID-19 variants [Table 2]. Higher percentages of respondents agreed that when viruses mutate, they can spread more efficiently (77.8%) and become more deadly (61.7%) and pathogenic (64.8%). More than half of respondents (57.4%) believe that people previously infected with COVID-19 may have partial immunity (i.e., natural immunity) against re-infection with the new variants. However, lower proportions have agreed on the effectiveness of the currently used vaccines against the new variants, with only 26.7% agreeing that these vaccines are totally effective and 53.7% agreeing that these vaccines are partially effective. Similar percentages agreed/disagreed on the efficacy of treatments currently used to treat new variants, with only 23.2% trusting the current treatments (as totally effective) and 50.4% agreeing on partial effectiveness. Half of the respondents (49.9%) still trust the currently employed diagnostics for new variants. However, 58.2% of respondents believe that testing antibody titer (concentration) is not decisive for determining if someone is immune or not against re-infection, while a complementary percentage (42.6%) believe otherwise (i.e. testing antibody titer is decisive). Regarding the evolution of VOCs, respondents agreed that the emergence of VOCs is attributed to both viral mutation mechanisms; antigenic shift (59.3%) and antigenic drift (64.3%).

**Table 2:** HCPs’ perceived knowledge about viral mutation and COVID-19 variants.

How much do you agree with the following statements about viral mutation and COVID-19 variants?			
	Agree (%)	Neutral (%)	Disagree (%)
When viruses mutate, they can spread more easily	329(77.8)	61(14.4)	33(7.8)
When viruses mutate, they become more deadly	261(61.7)	84(19.9)	78(18.4)
Mutant strains are more transmissible (infectious)	311(73.5)	68(16.1)	44(10.4)
When a virus mutates, it becomes more pathogenic (virulent)	274(64.8)	72(17.0)	77(18.2)
People previously infected with COVID-19 may be totally immune to the new variant strains of COVID-19.	122(28.8)	103(24.3)	198(46.8)
People previously infected with COVID-19 may be partially immune (i.e., less affected) to the new variant strains of COVID-19.	243(57.4)	91(21.5)	89(21.0)
New variants are diagnosed the same way as the original strain.	211(49.9)	82(19.4)	130(30.7)
Currently used COVID-19 Medicines are totally effective against new variants.	98(23.2)	125(29.6)	200(47.3)
Currently used COVID-19 Medicines are partially effective against new variants.	213(50.4)	111(26.2)	99(23.4)
Currently used COVID-19 Vaccines are totally effective against new variants.	113(26.7)	138(32.6)	172(40.7)
Currently used COVID-19 Vaccines are partially effective against new variants.	227(53.7)	115(27.2)	81(19.1)
New variants could evolve only through antigenic drift (i.e., the accumulation of mutations in the virus genes)	272(64.3)	101(23.9)	50(11.8)
New variants evolve through antigenic shift (i.e., recombination: the exchange of genetic material between two related viruses during co-infection of a host cell).	251(59.3)	111(26.2)	61(14.4)
Testing antibody titer (concentration) is decisive for determining if someone is immune or not against COVID-19 (i.e., viral infection).	180(42.6)	102(24.1)	141(33.3)
Testing antibody titer (concentration) is NOT decisive for determining if someone is immune or not because other immune components are involved in viral infections (i.e., natural killer cells).	246(58.2)	92(21.7)	85(20.1)

In assessing the respondent’s actual knowledge related to COVID-19 VOCs, Over half of respondents perceived all VOCs (compared to the original virus) as more transmissible (69.3%, 60.3%, 53.4% and 69%, for Alpha, Beta, Gamma and Delta variants respectively), and approximately half of respondents believed VOCs are more virulent (54.1%, 52%, 46.3% and 56.7% for Alpha, Beta, Gamma and Delta variants respectively), and Nearly half of the respondents thought that VOCs are related with higher mortality rates (48.5%, 46.6%, 45.4% and 54.6% for Alpha, Beta, Gamma and Delta variants respectively) [Table 3]. Furthermore, when asked about the efficacy of the current vaccines against VOCs, more than two thirds of respondents were either unsure about the current vaccines’ efficacy (31.2%, 38.3%, 41.4% and 32.2% for Alpha, Beta, Gamma and Delta variants respectively) or agreed that these vaccines would be ineffective (41.1%, 40.4%, 36.6% and 48% for Alpha, Beta, Gamma and Delta variants respectively).

**Table 3:** HCPs’ actual knowledge about viral mutation and COVID-19 variants.

Key Knowledge Questions: Which of the following statements regarding COVID-19 VOC in comparison with the original strain?	True (%)	Unsure (%)	False (%)	Correct answer
**Alpha variant: the UK or Kent variant (also known as B.1.1.7)**				
more transmissible	293(69.3)	84(19.9)	46(10.9)	**True**
cause more severe illness	229(54.1)	113(26.7)	81(19.1)	**True**
Have higher mortality rate	205(48.5)	134(31.7)	84(19.9)	**True**
Resistant to current vaccines	174(41.1)	132(31.2)	117(27.7)	**False**
**Beta variant; the South African variant (B.1.351)**				
more transmissible	255(60.3)	119(28.1)	49(11.6)	**True**
cause more severe illness	220(52.0)	128(30.3)	75(17.7)	**True**
Have higher mortality rate	197(46.6)	151(35.7)	75(17.7)	**True**
Resistant to current vaccines	171(40.4)	162(38.3)	90(21.3)	**True**
**Gamma variant; the Brazilian variant (P.1)**				
more transmissible	226(53.4)	144(34.0)	53(12.5)	**True**
cause more severe illness	196(46.3)	146(34.5)	81(19.1)	**True**
Have higher mortality rate	192(45.4)	156(36.9)	75(17.7)	**True**
Resistant to current vaccines	155(36.6)	175(41.4)	93(22.0)	**False**
**Delta variant; the Indian variant (B.1.617.2)**				
more transmissible	292(69.0)	84(19.9)	47(11.1)	**True**
cause more severe illness	240(56.7)	102(24.1)	81(19.1)	**True**
Have higher mortality rate	231(54.6)	115(27.2)	77(18.2)	**True**
Resistant to current vaccines	203(48.0)	136(32.2)	84(19.9)	**False**

**Fig 1:**
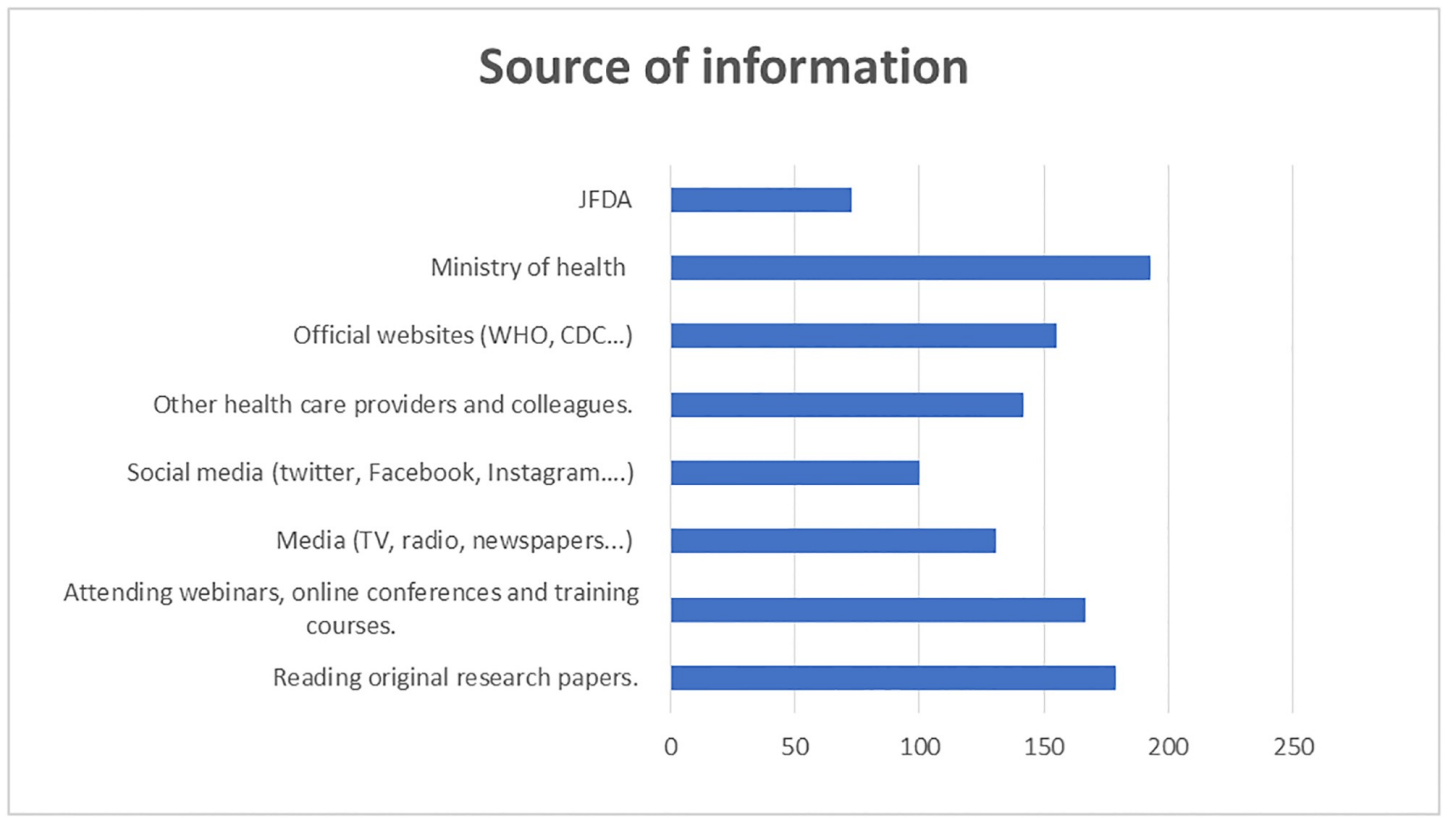
HCPs’ source of information about viral mutation and COVID-19 variants.

As shown in Fig 1, respondents mainly referred to the ministry of health (n = 193, 45.6%) as a source of information about the new COVID-19 VOCs, followed by reading original research papers (n = 179, 42.3%) and attending webinars, online conferences, and training (n = 167, 39.5%).

According to respondents, educating the public about the new COVID-19 VOCs was the responsibility of the Ministry of Health (MOH) (57.9%) and the National Committee for Epidemics (57.2%), followed by physicians (46.8%) [Table 4]. In addition, elderly people, patients with respiratory co-morbidities, and pregnant women were considered high-risk groups by 75.2%, 57.2%, and 53.7%, respectively, and should receive greater health care attention as they are more likely to be affected.

**Table 4:** HCPs’ perceived role towards viral mutation and COVID-19 variants.

Perceived role statement (check all that can apply)	Frequency (%)
**In your opinion, whose most responsible for educating the public about the new** **COVID-19 variants:**	
Physicians.	198(46.8)
Pharmacists.	112(26.5)
Nurses.	105(24.8)
Lab technicians.	47(11.1)
MOH	245(57.9)
JFDA	70(16.5)
the National Committee for Epidemics	242(57.2)
**Regarding the new COVID-19 variants, which of the following groups should get greater health care attention (people most likely affected)?**	
The elderly.	318 (75.2)
Children.	136(32.2)
Pregnant women.	227(53.7)
Patients with co-morbidities (hypertension, diabetes, rheumatoid arthritis, cancer, heart failure….).	166(39.2)
Smokers	115(27.2)
Patients with asthma, COPD, cystic fibrosis, or other respiratory diseases	242(57.2)
Healthy/athletic individuals.	42(9.9)
Patients previously infected with original COVID-19 strain.	45(10.6)
All populations should get the same attention	144(34.0)

Travel was not a source of worry among respondents, with only 16.8% being very or extremely worried about international travel [Table 5]. However, when asked about which countries they were more worried to travel into because of the new COVID-19 VOCs, 52.2% (n = 221) of respondents indicated that they would be worried to travel to India, followed by China (n = 142, 33.6%), African countries (n = 125, 29.6%), Brazil (n = 109, 25.8%) and the USA (n = 102, 24.1%). Lower percentages were worried to travel to other countries including UK (n = 64, 15.1%), European countries (n = 65, 15.4%), and the Middle East (n = 50, 11.8%).

**Table 5:** HCPs’ perceived risk and containment measures towards viral mutation and COVID-19 variants.

Statement					
**How much you will be worried about the following:**					
	Not at all Worried	Slightly Worried	Moderately Worried	Very Worried	Extremely Worried
International travel because of the new COVID-19 variants	96(22.7)	130(30.7)	126(29.8)	44(10.4)	27(6.4)
Getting the new COVID-19 VOCs at my work at the health facility	78(18.4)	114(27.0)	133(31.4)	63(14.9)	35(8.3)
My family members may get the new COVID-19 VOCs due to my work at the health facility	73(17.3)	79(18.7)	92(21.7)	104(24.6)	75(17.7)
My family are not supportive of me continuing my work at the health facility	154(36.4)	117(27.7)	83(19.6)	39(9.2)	30(7.1)
How much do you agree with the following statements regarding Health care provider’s attitudes towards the new COVID-19 variants? *					
	Strongly Agree	Agree	Neither agree nor disagree	Disagree	Strongly Disagree
Current precautions (wearing masks, hand washing, and social distancing) are sufficient for preventing the spread of the new COVID-19 variants	178(42.1)	161(38.1)	43 (10.2)	32 (7.6)	9 (2.1)
Tighter infection control measures and precautions should be applied to prevent the spread of the new COVID-19 variants.	173(40.9)	156 (36.9)	64 (15.1)	20 (4.7)	10 (2.4)
Partial or complete lockdown should be applied for preventing the spread of the new COVID-19 variants.	104(24.6)	89 (21.0)	90 (21.3)	71 (16.8)	69 (16.3)
People previously infected with COVID-19 still need to be vaccinated.	188(44.4)	140 (33.1)	63 (14.9)	22 (5.2)	10 (2.4)

More than half of the respondents were either slightly (27%) or moderately worried (31.4%) about getting the new COVID-19 VOCs at their work, whereas, 21.7% were moderately worried and 24.6% were very worried about their families getting the new COVID-19 VOCs from working at the healthcare facilities. [Table 5]. However, this was not shown to influence continuing the work at the health facility. Furthermore, around 40% of respondents strongly agreed that current precautions are sufficient for preventing the spread of the new COVID-19 VOCs. However, similar proportions strongly agreed that tighter infection control measures and precautions should be applied to prevent the spread of the new COVID-19 VOCs and that people previously infected with COVID-19 still need to be vaccinated.

## Discussion

COVID-19 pandemic disease continues to have a significant global threat to public health. Understanding the complexity of the COVID-19 situation and the significant role the healthcare professionals (including pharmacists) can help reduce the infection rates and control the transmission of COVID-19. This study explored perceptions, awareness, and attitudes of Jordanian health care professionals towards the new coronavirus SARS‐CoV‐2VOCs.

The healthcare professionals reported fair amount of knowledge about viral mutation and COVID-19 VOCs despite their primary sources of information about viral mutation and COVID-19 variants being the Jordan Ministry of Health, reading original research papers, and attending webinars, online conferences, and training courses. For example, most healthcare professionals agree that viruses can spread more quickly and become more deadly and pathogenic when they mutate. Also, a few healthcare professionals have agreed about the effectiveness of the currently used vaccines against the new VOCs, with only a quarter believing that vaccines are totally effective and more than half believing that vaccines are partially effective. Most healthcare professionals agreed that the new variants (the UK, South African, the Brazilian, and the Indian) are more transmissible, with about half agreeing that these variants can cause more severe illnesses. A fairly similar number of healthcare professionals believed that these variants have a higher mortality rate and are resistant to the current vaccines. Similar studies reported the same result [24,27,28]. For example, one cross-sectional study from Saudi Arabia reported that a low percentage of healthcare professionals have good knowledge about COVID-19 VOCs, which was related to the healthcare professional’s source of information being a mainly social network.

In assessing the respondents ‘actual knowledge related to COVID-19 VOCs, all VOCs were perceived as, compared to the original virus, more transmissible, more virulent, and related with higher mortality rates (which is more or less true according to the WHO definition of the VOCs). However, when asked about the efficacy of the current vaccines against VOCs, most respondents were either unsure about the current vaccines’ efficacy or agreed that these vaccines would be ineffective. This result is considered of particular concern as it might be one of the reasons behind the high rate of COVID-19 vaccine hesitancy among healthcare workers in specific [29] and public population in general [30].

The Jordanian ministry of health was the primary source of information the healthcare professionals referred to about the new COVID-19 VOCs. Other resources such as original research papers, webinars, and conferences were also used but to a lesser extent. Also, educating the public about the new COVID-19 VOCs was the responsibility of the Jordanian ministry of health, followed by physicians, pharmacists, and nurses. This was consistent with results from other studies [6,27,31–33]. For example, a recent cross-sectional Jordanian study reported that healthcare professional’s primary source of COVID-19 information is the Jordanian ministry of health and that physicians, pharmacists and nurses are responsible for educating the public [31]. However, a recent cross-sectional study from Saudi Arabia reported a contrasted result where healthcare professionals’ primary sources of COVID-19 information were social networks [27].

Healthcare professionals were not worried about travel, with only 16.8% being very or extremely worried about international travel. However, healthcare professionals were more worried about travelling into certain countries such as India, followed by China, African countries, Brazil, and the USA because of the new COVID-19 variants. The lowest percentages were reported with UK, European countries and the Middle East. In contrast, a Saudi cross-sectional study reported that healthcare professionals were highly worried about international travel, with the highest percentages associated with travel to the UK [24]. Most healthcare professionals were worried about their families getting the new COVID-19 VOCs from respondents who work at the health facility compared to that observed on themselves. However, this was not shown to affect continuing their work at the health facility. Similar studies reported the same result [23,24,27,33,34]. About less than half of the healthcare professionals strongly agreed that current precautions are sufficient for preventing the spread of the new COVID-19 VOCs. However, similar proportions strongly agreed that tighter infection control measures and precautions should be applied to prevent the spread of the new COVID-19 VOCs and that people previously infected with COVID-19 still need to be vaccinated.

This is the first Jordanian study that focuses solely on exploring healthcare professionals’ knowledge and perceptions of COVID-19 VOCs and the travel worries and restrictions caused by the COVID-19 VOCs and vaccine’s effectiveness against the new VOCs. This is significant as the findings from this study would guide future large-scale studies that may build on our findings. However, the small sample size with convenience sampling (although the sampling technique was based on the study’s objectives during this ongoing pandemic) was potentially limiting the representation of the study’s sample. Therefore, the study findings should be interpreted carefully. Although a convenience sample was used for this study, this may have not affected the results particularly as the obtained sample was fairly representative of HCPs in Jordan [26], given that; gender wise; there are more female healthcare professionals in Jordan (more females were included in the study sample (67.8%) than males), also, residency wise, more healthcare professionals are situated in the middle governorates, compared to north and south (76.8% of respondents were from the middle governorates). In addition, nurses and midwives comprise the largest sector of HCPs in Jordan, followed by physicians and pharmacists (nurses/ midwives were the most frequent health care professionals in the study (35.9%), followed by pharmacists (30.0%). Furthermore, more HCPs work in the private sector than in public sector (52.7% of respondents were from the private sector).

Moreover, responses were collected mainly from COVID-19 frontline physicians, pharmacists, and nurses and did not include other healthcare professionals such as dentists and laboratory professionals, which may lead to selection bias. Finally, the online questionnaire nature possibly leads to having a voluntary response and non-response bias; however, this limitation was reduced by using a paper-based questionnaire along with the online questionnaire.

## Conclusion

This is the first Jordanian study examining healthcare professionals’ perceptions and knowledge about the new COVID-19 VOCs (in terms of emergence, transmissibility, pathogenicity, and vaccine resistance) and their travel worries based on these new variants. This study showed that the healthcare professionals have fair and satisfactory knowledge and perception towards the new COVID-19 VOCs and they were not having travel worries except for India, China, Africa, Brazil, and the USA. Findings from this study would guide policymakers to scale up educational efforts to disseminate reliable information on the different variants and provide recommendations about receiving a vaccine booster. As the new COVID-19 variants evolve, healthcare professionals’ knowledge and perceptions will likely change. Therefore, future large-scale studies should provide more insight into the new COVID-19 VOCs.

## Supporting information

S1 Appendix (DOCX)
