# Supplementary figures and images for: Assessment of Jordanian health care professionals’ perception towards new COVID-19 variants of concern
Source: PLoS One. 2022 Nov 18;17(11):e0265797. doi: 10.1371/journal.pone.0265797 (PMC9674127; doi:10.1371/journal.pone.0265797)

**Appendix 1**

**
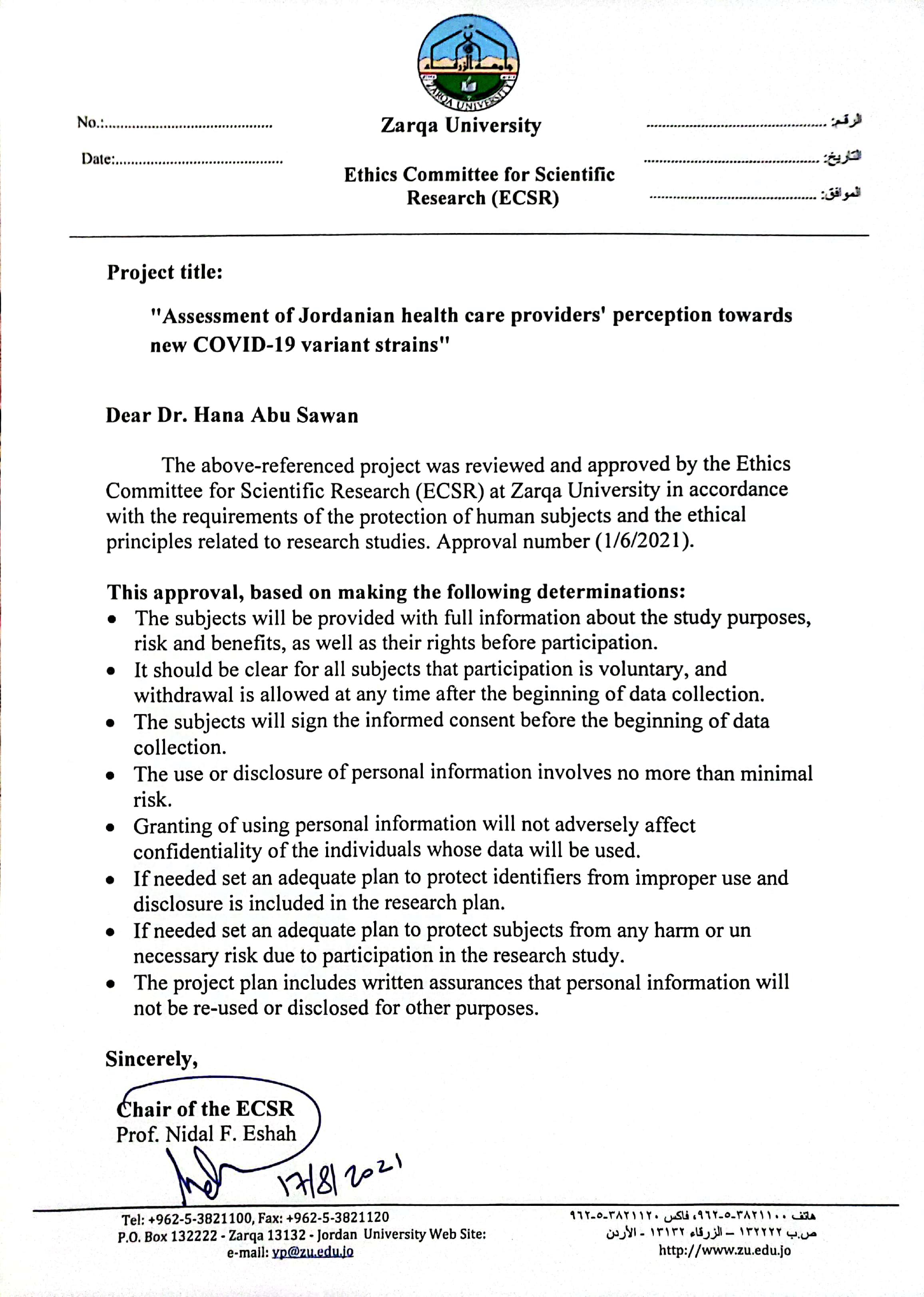
**

Supplement: S1 Appendix — (DOCX) [file pone.0265797.s001.docx]
